# The Place of Psychotherapy in Contemporary Psychiatry

**Published:** 2014

**Authors:** Saman Tavakoli

**Affiliations:** 1 Psychiatrist, Iranian Psychiatric Association, Tehran, Iran.

## Abstract

Psychotherapy has long been an essential component of clinical psychiatry and many young physicians choose to train in psychiatry residency programs in order to acquire necessary knowledge and skills, and become competent psychotherapists. Recent advances in psychopharmacology and neuroscience, and growing dominance of managed care and evidence-based medicine have had dramatic impacts on health care delivery systems and clinical psychiatry practice. Despite these changes in the field of mental health, psychotherapy still remains a crucial part of clinical psychiatry and comprises a great proportion of psychiatrists’ clinical practice. Hence, accreditation agencies and regulatory bodies determine compulsory minimum requirements for psychiatry residency programs to ensure that residents, at the end of their specialty training, can demonstrate competence in managing their patients through applying different approaches of psychotherapy.

At the turn of the 20^th^ century, Sigmund Freud, an eminent Austrian neurologist, developed psychoanalysis as “a procedure for the investigation of mental processes”, “a method for the treatment of neurotic disorders”, and “a collection of psychological information obtained along those lines” (1). During the first half of the century, psychoanalysis became the dominant paradigm in psychiatry and the main approach for the treatment of psychiatric disorders. Following the introduction of psychiatric medications in the next decades, however, the situation changed. In less than a decade after the identification of lithium’s sedative effects and its efficacy in the treatment of manic patients by John Cade in 1949, prototype drugs for other mental conditions were also discovered. Chlorpromazine (an antipsychotic), iproniazid (a monoamine oxidase inhibitor), imipramine (a tricyclic antidepressant), and chlordiazepoxide (a benzodiazepine) were all identified within less than 10 years. Pharmacological treatments for major psychotic, mood, and anxiety disorders were developed by the end of the 1950s (2). The achievements gained during 1945-1957 not only revolutionized the biological treatment of psychiatric disorders, but also attracted attention to the role of neurotransmitters in the etiology of such disorders. Subsequent to the mentioned achievements, efforts were made, particularly during the second half of the 20^th^ century, to develop biological models to explain psychiatric disorders and to propose novel psychiatric medications. In other words, psychiatry, which used to endorse ‘soft’ bases of interpersonal relationships, shifted toward a ‘hard’ scientific approach in which physicochemical models were expected to explain psychiatric disorders. The ‘hard’ scientific approach assumes that such models can simply be assessed through scientific and empirical methods and are thus more reliable than models using ‘soft’ bases. It also argues that applied physics and chemistry can ultimately resolve all problems in the world, including psychiatric disorders (3). Therefore, in order to consider a body of knowledge as scientific and reliable or to administer a particular treatment modality for psychiatric disorders, rigorous methodologies have to be applied to validate that knowledge or determine the efficacy of those treatment methods. In this context, understanding the position of psychotherapy in contemporary psychiatry would be more complex. In efforts to resolve the problem of empirical support for psychotherapies, evidence has been provided by the application of acceptable methodologies in numerous studies on the efficacy of some short-term manualized therapies, e.g. cognitive-behavioral therapy, in the treatment of various psychiatric disorders. Nevertheless, common research methods in medicine and randomized controlled trials (RCTs), as the ‘gold standard’ of evidence, cannot be easily used to evaluate psychotherapies, particularly long-term psychodynamic psychotherapy, or those dealing with a wide range of symptoms and problems (e.g. personality disorders). Some scholars, however, contend that research to evaluate the efficacy of psychotherapeutic methods is more challenging and complex, but essentially not less possible, than that in other fields of medicine. Methodological complexities, the required time and cost for such research, and pharmaceutical companies’ refusal to invest in psychotherapeutic research limit the number of studies on the efficacy of psychotherapies, particularly long-term and dynamic psychotherapies (4, 5). Some believe that for psychotherapy, as a psychiatric practice, to survive under the dominance of evidence-based medicine, researchers should insist on the inapplicability of common efficacy evaluation methods and RCTs to psychotherapy, and also define appropriate criteria and methods to assess the usefulness of psychotherapies (6).

Despite the development of various medications and drug classes over the recent decades, the new drugs do not seem to be more effective than their ancestors. In fact, none of the novel antidepressants can supersede the first generation of antidepressants (such as imipramine and monoamine oxidase inhibitors) introduced in the 1950s. Consequently, many patients gain modest benefits or none at all. Likewise, the antipsychotic efficacy of clozapine (discovered in 1960) has not been surpassed by any new drugs. Although numerous mood-stabilizing drug classes have been introduced during the past years, lithium is still the standard treatment for bipolar disorder (2). Meanwhile, there is no pharmacological treatment with documented efficacy for the core symptoms and psychopathology of some disorders such as social and language deficits in autism, negative symptoms of schizophrenia, and core psychopathological elements of personality disorders. In other words, despite the advances in psychopharmacology, the role of psychotherapy in understanding the etiology and treatment of psychiatric disorders cannot be neglected. Moreover, it is recommended in various clinical practice guidelines for most psychiatric disorders as a single treatment or in combination with other therapeutic modalities (7). 

In their introduction to the American Psychiatric Publishing Textbook of Clinical Psychiatry, Gabbard et al. write about a dichotomy due to the existence of conflicting views in psychiatry (5). Based on this dichotomous perspective, while psychotherapy was applied for problems with psychological origins, disorders and conditions originating from the human brain were to be treated with biological and pharmacological treatments (8). However, based on later studies, especially those performed by Eric Kandel, psychotherapies act at the synaptic level in the brain and psychotherapeutic process could be described as an environmental experience influencing gene expression in patients (8). In other words, similar to pharmacological treatments, psychotherapies tend to help patients by affecting the brain. According to Lipowski, “after a period of ‘brainless’ psychiatry during which the focus was merely on psychodynamic and social factors, the attention was shifted toward contrasting ideas such as radical biologism and ‘mindless’ psychiatry” (9). These dichotomous perspectives are currently supplanted by a more balanced view which considers psychotherapy as a basic science -like biochemistry and anatomy- applicable to all fields of psychiatry (9). Furthermore, psychotherapy can be defined as a biological treatment that helps patients by causing particular changes in their brain.

***Psychotherapy in Psychiatric Practice***

Despite the remarkable progress in the development and application of pharmacological treatments for psychiatric disorders, psychotherapy is still playing a critical role in the treatment of psychiatric patients. While about 3% of Americans receive psychotherapy from their psychiatrists, psychologists, and social workers per annum (10), psychiatrists have a unique place in the treatment of psychiatric patients, as they are the only group of mental healthcare providers capable of prescribing medicines and providing integrative pharmacotherapy and psychotherapy. Since combining medication prescription with psychotherapy is a major characteristic of current psychiatric practice, competency in conducting such care is a key component of the required training for psychiatrists. Surveys conducted in countries such as the USA and Canada show that psychiatrists spend a substantial amount of their clinical practice providing psychotherapy (11). Olfson et al. found that “psychotherapy was received by 79% of the patients visiting psychiatrists” (11). Similarly, a previous study reported that 92% of psychiatrists spend about half of their time in clinical practice performing psychotherapy (11). Following recent changes in reimbursement policies and the insurance companies’ willingness to cover brief medication management visits, particularly in the USA, researchers have increasingly focused on trends of psychotherapy practice by psychiatrists. Mojtabai and Olfson evaluated the national trends in psychotherapy provision by psychiatrists in their offices in the USA. They reported a decline in the visits involving psychotherapy by psychiatrists, from 44.4% in 1996-1997 to 28.9% in 2004-2005 (7).

In other words, although psychotherapy occupies a great proportion of psychiatrists’ time, the availability of psychiatric medications with few side effects, managed care, reimbursement policies, and the insurance companies’ inclination to cover brief Med Checks have all contributed to the reduction in psychotherapy provision by psychiatrists in recent years (7). Furthermore, the growing number of patients with mental health care demands along with the limited number of psychiatrists results in long waiting lists. Financial factors are also partly responsible for the observed trend. In fact, psychiatrists earn substantially greater income from brief Med Checks than from psychotherapy. For instance, in the USA, over a 45-50-minute period, a psychiatrist can manage three 15-minute brief medication management visits and earn $150. However, devoting the same time to psychotherapy would yield $90 (7). Studies show that patients who paid treatment costs out of pocket had the highest chance of receiving psychotherapy. Moreover, patients with private insurance were more likely to receive psychotherapy than those covered by public insurance (7). One interesting finding was that over 40% of psychiatrists provided psychotherapy for either all or none of their patients. This finding suggests an ideological dichotomy in contemporary psychiatry, i.e. a large group of psychiatrists belong to one of the psychotherapy or the pharmacotherapy camps (7).

Based on surveys on psychiatric practice, one can conclude that despite the recent reduction in psychotherapy provision by psychiatrists, this method of care is still a major part of clinical psychiatry. Developing competency in psychotherapy provision should thus be regarded as an indispensable component of psychiatric training.

***Psychotherapy in Psychiatric Training***

As discussed earlier, psychotherapy is a defining characteristic of psychiatrists' career (4). Surveys have demonstrated that psychiatrists devote a considerable proportion of their clinical practice time to psychotherapy (11). On the other hand, acquiring the knowledge and skills required for psychotherapy is one of the major reasons young physicians choose to study psychiatry (12). In a study by Hadjipavlou and Ogrodniczuk, a large proportion of psychiatry residents reported the chance to learn and practice psychotherapy as an important factor for selecting psychiatry as their future career. Moreover, they introduced their competency in psychotherapy as a crucial part of their professional identity (13). In the era of health care reform and the dominance of managed care, evidence-based medicine, and reimbursement systems based on brief Med Checks, psychiatric residency training has changed to embrace both long-term psychodynamic therapies and short-term, evidence-based psychotherapies (14). Although training on dynamic psychotherapy remains a key component of psychiatry training, acquiring competency in other approaches is also required. Therefore, accreditation authorities and organizations generally oblige psychiatry residency programs to provide their residents the opportunity for the development of competency in various psychotherapy approaches (14).

The Accreditation Council for Graduate Medical Education (ACGME) program requirements for graduate medical education in psychiatry (Effective July 1, 2014) imply that psychiatry residents should be trained to acquire competence in “applying supportive, psychodynamic, and cognitive-behavioral psychotherapies to both brief and long-term patient encounters, as well as to ensuring exposure to family, couples, group, and other individual evidence-based psychotherapies” (15). Likewise, the Royal College of Psychiatrists in the UK considers learning different types of psychotherapy including “brief therapy, cognitive behavioral therapy, psychodynamic therapy, psychotherapy combined with psychopharmacology, supportive therapy and all delivery systems of psychotherapy (that is individual, group, and family)” to be essential for psychiatric trainees (16). Moreover, individuals who have completed their psychiatry core training can attend different fields of advanced training, including psychotherapy. After acquiring the necessary competencies, the participants of advanced training in psychotherapy are identified as ‘specialists in psychotherapy’ (17).

Similar models of psychiatric training also exist in other countries. Based on residency curricula and programs in various countries, actual psychiatric practice, and the competencies required for psychiatrists, psychotherapy training can be considered as an indispensable part of psychiatric residency training. In fact, from the beginning of their training, psychiatry residents learn to obtain a psychotherapeutic understanding of their patients, in the inpatient and outpatient settings, and have opportunities to use integrated treatments, combining pharmacological treatments and psychotherapy, to help the patients. In addition, during their psychotherapy training, the residents are compelled to attend particular theoretical courses and have direct clinical experience under close supervision of experienced faculty members. Such training provides the residents with clinical experience and helps them acquire the competencies necessary for different approaches of psychotherapy, including cognitive-behavioral, psychodynamic, and supportive psychotherapy. These approaches are practiced in different treatment settings such as individual, group, family, and couple therapies. However, different levels of expected competency are specified for the residents; while they are obligated to become competent in some cases, e.g. individual therapy of non-complicated patients through different approaches, they may only observe group and family therapies, based on the available resources of residency programs. Residents who are more interested in psychotherapy will find ways to gain maximum experience from the available facilities.

## Conclusion

Psychotherapy is an essential part of the professional identity of psychiatrists. Many young physicians prefer to enroll in graduate medical education in psychiatry in order to learn and practice psychotherapy in their future career. Despite advances in biological psychiatry, neuroscience, and pharmacotherapy, psychotherapy is still recommended in clinical practice guidelines for different psychiatric disorders. Moreover, despite the fluctuations in the relation between psychiatry and psychotherapy, a great proportion of psychiatrists’ time is devoted to provision of psychotherapy. Considering the dominance of biological psychiatry and neuroscience, development of various psychiatric medications, debates over the cost-effectiveness of different methods, and emphasis on evidence-based treatments in managed care-based health systems, contemporary psychotherapy is compelled to bridge its own concepts and methods with new findings, especially in the field of neuroscience. More studies with rigorous methodologies are also warranted to show the efficacy of various psychotherapeutic approaches in the treatment of different psychiatric disorders.

In the absence of adequate systematic information on the pattern of psychiatric training and practice in Iran, numerous surveys are needed to determine the level and pattern of psychotherapy practices by psychiatrists both in their offices and in hospitals. According to the latest approved national psychiatry curriculum (18, 19), a substantial part of psychiatry residency training is dedicated to psychotherapy training, and it is expected that residents become competent in applying different psychotherapy approaches. Nevertheless, further studies are required to provide systematic information about the domains, methods, and depth of psychotherapy training, and the expected level of competency at the end of the residency training in different programs. Such data on involvement of psychiatrists and other mental healthcare providers in providing psychotherapy in their clinical practice along with information on the current situation of psychotherapy training in these groups would undoubtedly be beneficial and necessary for future planning.

## Notes

**Declaration of interest:** None.
